# Long noncoding RNA NEAT1 is involved in the protective effect of Klotho on renal tubular epithelial cells in diabetic kidney disease through the ERK1/2 signaling pathway

**DOI:** 10.1038/s12276-020-0381-5

**Published:** 2020-02-14

**Authors:** Yan-Lin Yang, Meng Xue, Yi-Jie Jia, Fang Hu, Zong-Ji Zheng, Ling Wang, Ze-Kun Si, Yao-Ming Xue

**Affiliations:** 1 Department of Endocrinology and Metabolism, Nanfang Hospital, Southern Medical University, Guangzhou, Guangdong China; 2 Department of Endocrinology and Metabolism, Shenzhen People’s Hospital, Second Affiliated Hospital of Jinan University, Shenzhen, Guangdong China; 3 Department of Endocrinology and Metabolism, The Fifth Affiliated Hospital Sun Yat-Sen University, Zhuhai, Guangdong China

**Keywords:** Mechanisms of disease, Long non-coding RNAs, Kidney, Transdifferentiation

## Abstract

Klotho, an antiaging protein, has been shown to play a protective role in renal tubular epithelial-mesenchymal transition (EMT) during the development of diabetic kidney disease (DKD). Long noncoding RNAs (lncRNAs) participate in the progression of EMT in many diseases. However, the effect of Klotho on lncRNAs during the development of DKD is still unknown. In this study, we found that Klotho overexpression in high-fat diet (HFD)- and streptozotocin (STZ)-induced DKD mice significantly inhibited the expression of lncRNA nuclear-enriched abundant transcript 1 (Neat1). We demonstrated that NEAT1 was significantly upregulated in both bovine serum albumin (BSA)-stimulated HK2 cells and mice with HFD- and STZ-induced diabetes. In addition, we observed that Klotho displays colocalization with NEAT1. Furthermore, overexpression of Klotho can inhibit the high expression of NEAT1 in BSA-stimulated HK2 cells, while silencing Klotho can further upregulate the expression of NEAT1. Silencing NEAT1 in HK2 cells resulted in inhibition of the EMT-related markers alpha smooth muscle actin (α-SMA) and vimentin (VIM) and the renal fibrosis-related markers transforming growth factor-β1 (TGF-β1) and connective tissue growth factor (CTGF). The effect of NEAT1 on DKD was partly mediated by regulation of the ERK1/2 signaling pathway. Finally, we found that silencing NEAT1 can reverse the activation of EMT and fibrosis caused by Klotho silencing in a manner dependent on the ERK1/2 signaling pathway. These findings reveal a new regulatory pathway by which Klotho regulates ERK1/2 signaling via NEAT1 to protect against EMT and renal fibrosis, suggesting that NEAT1 is a potential therapeutic target for DKD.

## Kidney disease: Effect of an anti-aging protein

An anti-ageing protein called Klotho helps protect against kidney failure in mice and human cells by silencing a long non-coding RNA molecule. The regulatory RNA involved, known as NEAT1, promotes cellular transformations associated with the disease process. A team led by Yao-Ming Xue from Southern Medical University in Guangdong, China, showed that levels of NEAT1 are elevated in mouse models of diabetic kidney disease and in injured human kidney calls. The identification of NEAT1 in kidney disease thus provides a novel therapeutic target. After demonstrating that Klotho and NEAT1 interact directly with each other in cells, they experimentally boosted Klotho expression and observed suppressed levels of NEAT1. As a consequence, the cells displayed lower levels of the proteins linked to the progressive deposition of fibrosis in the kidneys.

## Introduction

With the rising prevalence of diabetes mellitus in recent decades, diabetic kidney disease (DKD) has become more common than glomerulonephritis nephropathy in China^1^. Notably, 40–45% of patients with type 1 diabetes mellitus (T1DM) develop DKD and progress to end-stage renal disease (ESRD) or die before its onset^2^. Increasing evidence suggests that renal tubules play a causative role in the progression of DKD^3^. Tubulointerstitial fibrosis is one of the most common pathological changes associated with renal tubules in DKD. Some studies have reported that the extent of the interstitial fibrosis and tubular atrophy (IFTA) score is a significant predictor of renal prognosis in advanced DKD^4^. In recent years, we have reported that the epithelial-to-mesenchymal transition (EMT) of renal tubular epithelial cells promotes tubulointerstitial fibrosis during the process of accelerating DKD progression^5,6^. Albumin is a key factor for promoting EMT in renal tubular epithelial cells in DKD^6,7^. Therefore, it is of great significance to explore the pathophysiological mechanism in the renal tubules during the progression of DKD.

Klotho, an antiaging protein, is highly expressed in renal tubular tissue and is recognized as a promising protein that provides a basis for antifibrotic treatment strategies^8^. Recently, studies have confirmed that decreased Klotho levels in the early stage of T2DM can predict renal function decline^9^. Klotho deficiency exacerbates early tubulointerstitial fibrosis in DKD mice, and recombinant Klotho therapy can significantly improve renal function and renal fibrosis^10–12^. Our previous report found that the preventative effects of Klotho against renal fibrosis are partially attributable to the downregulation of Egr-1 expression by inhibiting TGF-β1/Smad3 signaling in high-glucose-treated human MCs^13^. However, the precise molecular mechanisms by which Klotho regulates EMT and tubulointerstitial fibrosis during the progression of DKD are largely unknown.

Long noncoding RNA was initially considered to be “transcriptional noise,” but recently, some studies have argued that it has many functions in various pathophysiological processes^14^. Increasing evidence suggests that lncRNAs are regulators of almost every cellular process, including the proliferation, differentiation and apoptosis of cells, and the expression of these noncoding molecules is strictly regulated under physiological conditions as well as in several human diseases^15^. In recent years, lncRNAs have rapidly emerged as key regulators of EMT in a variety of organ fibrosis-related diseases, such as the lncRNAs MALAT1, H19, HOTAIR, and NEAT1^16^. In particular, the effect of lncRNA NEAT1 in DKD has not been reported.

Interestingly, Neat1 was one of the lncRNAs with the most significant expression changes in our Klotho-overexpressing DKD mice in this study. Thus, further exploration of the mechanism by which Neat1 is involved in the renal protection mediated by Klotho provides a theoretical basis for Klotho treatment of DKD. We also explored the mechanism of NEAT1-regulated EMT and tubulointerstitial fibrosis in DKD to identify new possible targets for the treatment of DKD.

## Materials and methods

### Animal modeling and grouping

Three- to four-week-old male C57BL/6 J mice were purchased from Guangdong Medical Laboratory Animal Center and randomly divided into a control group (*n* = 5) and a DM model group (*n* = 15). The model of DM in mice was established according to our previous research methods^17,18^. In brief, the control mice were fed a normal diet, while the DM mice were fed a high-fat diet (HFD; protein 20%, fat 60%, carbohydrate 20%, D12492, Guangdong Medical Laboratory Animal Center) for 4 weeks. After that, the mice were fasted for 12 h before receiving an intraperitoneal injection of streptozotocin (STZ; 120 mg/kg in citrate buffer, pH = 4.5, Sigma, USA). The control mice were injected with sodium citrate buffer. The two groups continued to be fed a normal diet or a HFD until the end of the experiment. After 1 week of STZ injection, a random blood glucose level > 16.7 mmol/L indicated successful establishment of the diabetes model in the mice. We randomly divided the DM mice into the DM group (*n* = 5), DM + pCMV-vector group (*n* = 5), and DM + pCMV-Klotho group (*n* = 5) after the diabetes model was established for 12 weeks. Mice in the control group and the DM group were sacrificed. Mice in the DM + pCMV-vector group and DM + pCMV-Klotho group were transfected with plasmids by hydrodynamic transfection in vivo. Briefly, the mice were rapidly injected with a large volume of pCMV-vector or pCMV-Klotho plasmid solution through the tail vein once a week for 4 weeks^19^. The animal experiments were conducted in accordance with the NIH Experimental Animal Management guidelines and approved by the committee of the Laboratory Animal Center, Nanfang Hospital, Southern Medical University, Guangzhou, China (certificate number: SYXK2015-0056).

### Serum biochemical parameters and urinary albumin

Blood samples were collected from the orbital sinus after intraperitoneal injection of pentobarbital sodium and fasting for 8 h. Triglycerides (TG), total cholesterol (TC), low-density lipoprotein (LDL) cholesterol and glucose were measured by ELISA (Shanghai Fanke Biotechnology Co., Ltd.) according to the manufacturer’s instructions. 24 h urine samples were collected in metabolic cages. Urinary albumin was measured by ELISA (Bethyl Laboratories Inc., Montgomery, TX, USA) according to the manufacturer’s instructions.

### Kidney histopathology and immunohistochemistry

The mouse kidney tissues were first fixed with 4% paraformaldehyde for 12 h, embedded in paraffin and cut into 3-μm-thick sections. The renal sections were stained with hematoxylin and eosin (H&E) and Masson’s trichrome as previously described^20,21^. Histopathological examination was then performed under light microscopy at a magnification of 400×. The expression of TGF-β1, CTGF, α-SMA and Klotho in the renal tissue was detected by immunohistochemistry as described previously^20,21^. Briefly, renal sections were incubated with rabbit polyclonal anti-TGF-β1 (1:150, Santa Cruz, USA), mouse monoclonal anti-CTGF (1:200, Santa Cruz, USA), mouse monoclonal anti-α-SMA (1:500, Boster, China) and goat polyclonal anti-Klotho (1:500, R&D, USA) antibodies overnight at 4 °C, followed by incubation with a horseradish peroxidase-conjugated corresponding secondary antibody. Hematoxylin was applied as a counterstain. Finally, the stained sections were examined using light microscopy of randomly selected sections at a magnification of ×400. Immunohistochemical staining was semiquantified with ImageJ software.

### Cell culture and transfection

The human proximal tubular cell line (HK-2) was obtained from the Cell Bank of the Type Culture Collection (Chinese Academy of Sciences, Shanghai, China). The cells were cultured with RPMI‐1640 medium (Invitrogen, Shanghai, China) containing 10% fetal bovine serum (FBS; Gibco, Australia) in a 5% CO_2_ incubator at 37 °C. Before intervention, the cell growth cycle was synchronized by starvation treatment with RPMI-1640 medium containing 2% FBS for 12 h. Bovine serum albumin (BSA) was used to stimulate HK-2 cells to mimic DKD in vitro. The pcDNA3-Klotho plasmid (Addgene plasmid # 17712, USA) and si-Klotho (RiboBio, Guangzhou, China) were used to overexpress and knockdown Klotho in HK-2 cells, respectively. The GV417-NEAT1 plasmid (Gene, Shanghai, China) and si-NEAT1 (GenaPharma, Shanghai, China) were used to overexpress and interfere with the expression of lncRNA NEAT1 in HK-2 cells, respectively. The sequences of si-Klotho and si-NEAT1 are shown in Table 1. All transfections were conducted with Lipofectamine® 3000 (Invitrogen, Carlsbad, CA, USA) according to the manufacturer’s instructions.

**Table 1:** Sequences of siRNAs.

siRNA	Sequences
si-Klotho	5’-GCAGAATTACATAAACGAA-3’
si-NC	sense 5’-UUCUCCGAACGUGUCACGUTT-3’ antisense 5’-ACGUGACACGUUCGGAGAATT-3’
si-NEAT1-1	sense 5’-GGAGGGCUAAUCUUCAACUTT-3’ antisense 5’-AGUUGAAGAUUAGCCCUCCTT-3’
si-NEAT1-2	sense 5’-GCCAUCAGCUUUGAAUAAATT-3’ antisense 5’-UUUAUUCAAAGCUGAUGGCTT-3'
si-NEAT1-3	sense 5’-GUUGGUCAUUCCUAAAUCUTT-3’ antisense 5’-AGAUUUAGGAAUGACCAACTT-3’
si-NEAT1-4	sense 5’-GAACUUUACUUCGUUAGAUTT-3’ antisense 5’-AUCUAACGAAGUAAAGUUCTT-3’

### RNA isolation and quantitative real-time polymerase chain reaction (qRT-PCR)

Total RNA was isolated from renal tissues and HK-2 cells using TRIzol reagent (Takara, Dalian, China) according to the manufacturer’s protocols. The RNA concentration and purity were detected by a NanoDrop ND-1000 spectrophotometer (Thermo Fisher Scientific, Wilmington, DE, USA). Reverse transcription was performed with PrimeScript^TM^ RT Master Mix (Takara, Dalian, China). qRT-PCR was conducted in a Roche LightCycler 480 Real‐Time PCR System (Roche, Basel, Switzerland) with SYBR® Premix Ex Taq™ (Takara, Dalian, China). The primer sequences are listed in Table 2. The expression of each gene was calculated using the 2−ΔΔCt method, and GAPDH was used as an internal control.

**Table 2:** Sequences of primers used for qRT-PCR.

Gene	Primers	
GAPDH	forward	5’-GAACGGGAAGCTCACTGG-3’
	reverse	5’-GCCTGCTTCACCACCTTCT-3’
H-Klotho	forward	5’-GAAAAATGGCTTCCCTCCTT-3’
	reverse	5’-ACAACTCCCCAAGCAAAGTC-3’
H-NEAT1	forward	5’-CCTGCCTTCTTGTGCGTTTC-3’
	reverse	5’-CTTGTACCCTCCCAGCGTTT-3’
H-TGF-β1	forward	5’-GCTAAGGCGAAAGCCCTCAAT-3’
	reverse	5’-CCTGGCGATACCTCAGCAACC-3’
H-CTGF	forward	5’-CTTGCGAAGCTGACCTGGAA-3’
	reverse	5’-AGCTCAAACTTGATAGGCTTGGAG-3’
H-VIM	forward	5’-CGTGATGCTGAGAAGTTTCGT-3’
	reverse	5’-TGGATTCACTCCCTCTGGTTG-3’
H-α-SMA	forward	5′‐TACTACTGCTGAGCGTGAGA‐3′
	reverse	5′‐CATCAGGCAACTCGTAACTC‐3′
M-Klotho	forward	5’-TTGGGTCACTGGGTCAATCTC-3’
	reverse	5’-CCGGCACGATAGGTCATGTT-3’
M-Neat1	forward	5’-CTGGTTTATCCCAGCGTCAT-3’
	reverse	5’-CTTACCAGACCGCTGACACA-3’
M- TGF-β1	forward	5’-CCACCTGCAAGACCATCGAC-3’
	reverse	5’-CTGGCGAGCCTTAGTTTGGAC-3’
M-CTGF	forward	5’-TTCCCGGGTGTAAGCAAGAA-3’
	reverse	5’-TGAAGTATGCTGGGGGTTGG-3’
M-Vimentin	forward	5’-GCCTCTGCCAACCTTTTCTTCC-3’
	reverse	5’-GTCCATCTCTGGTCTCAACCGTCT-3’
M-α-SMA	forward	5’-GAGGCACCACTGAACCCTAA-3’
	reverse	5’-CATCTCCAGAGTCCAGCACA-3’

### Western blot analysis

The total protein of HK-2 cells was extracted with cold RIPA lysis buffer (KeyGEN Biotech, China), and the protein concentration was detected with a BCA assay (Takara, Dalian, China). Proteins (50 μg) with different molecular weights were isolated by 10% sodium dodecyl sulfate polyacrylamide gel electrophoresis (SDS-PAGE; Bio-Rad, Hercules, CA, USA) and electrotransferred to polyvinylidene fluoride membranes (PVDF; Merck Millipore, MA, USA). The membranes were blocked at room temperature for 1 h with 5% skim milk before being incubated at 4 °C overnight with primary antibodies specific for Klotho (1:300, R&D, USA), TGF-β1 (1:400, Santa Cruz, USA), CTGF (1:200, Santa Cruz, USA), VIM (1:200, Santa Cruz, USA), α-SMA (1:300, Boster, China), ERK1/2 (1:300, Cell Signaling Technology, USA) and GAPDH (1:1000, ProteinTech, China). Next, the membranes were incubated with the corresponding fluorescent secondary antibodies (1:15,000; LI-COR Biosciences, Lincoln, NE, USA) at room temperature for 1 h and visualized with an Odyssey infrared imaging system (LI-COR, Lincoln, NE, USA). Finally, the images were semiquantified using ImageJ software.

### Immunofluorescence and fluorescence in situ hybridization (FISH) in HK-2 cells

Slides of HK-2 cells were fixed with 4% paraformaldehyde for 20 min and processed by a combined immunofluorescence and FISH protocol, which was developed for the simultaneous detection of Klotho and NEAT1^22^. First, the slides were hybridized with 8 ng/μl NEAT1 probes (Ribobio, Guangzhou, China) at 37 °C overnight. Subsequently, the slides were incubated with anti-Klotho (1:25, Santa Cruz, USA) at 4 °C overnight. Then, the reaction was developed with FITC goat anti-mouse IgG (1:100, ProteinTech, China) for 1 h. Finally, nuclei were counterstained with DAPI. The slides were visualized for immunofluorescence and FISH with a fluorescence microscope at a magnification of ×400.

### RNA immunoprecipitation (RIP)

RNA immunoprecipitation (RIP) analysis was performed with a BersinBio^TM^ RIP Kit according to the manufacturer’s instructions. Briefly, HK-2 cells were collected by washing, scraping, centrifuging and lysing in lysis buffer. Cell lysates were added to magnetic beads and incubated on an end-to-end rotator with anti-Klotho antibody (3 μg, Santa Cruz, USA) at 4 °C overnight. Anti-IgG was used as a normal control (3 μg, ABclonal, China). The samples were washed twice and treated with buffer containing proteinase K. Immunoprecipitated RNA was isolated with TRIzol reagent (Takara, Dalian, China), and the expression of lncRNA NEAT1 was detected by qRT-PCR as described above.

### Statistical analyses

All results were analyzed with SPSS 20.0 software. All data are presented as the mean ± SEM. Student’s *t*-test and one-way ANOVA were used to determine statistical significance. Bonferroni’s and Dunnett’s T3 tests were used to perform multiple comparisons. A *p*-value < 0.05 was considered statistically significant.

## Results

### Klotho regulates lncRNA Neat1 expression in DM mice

**Fig. 1:**
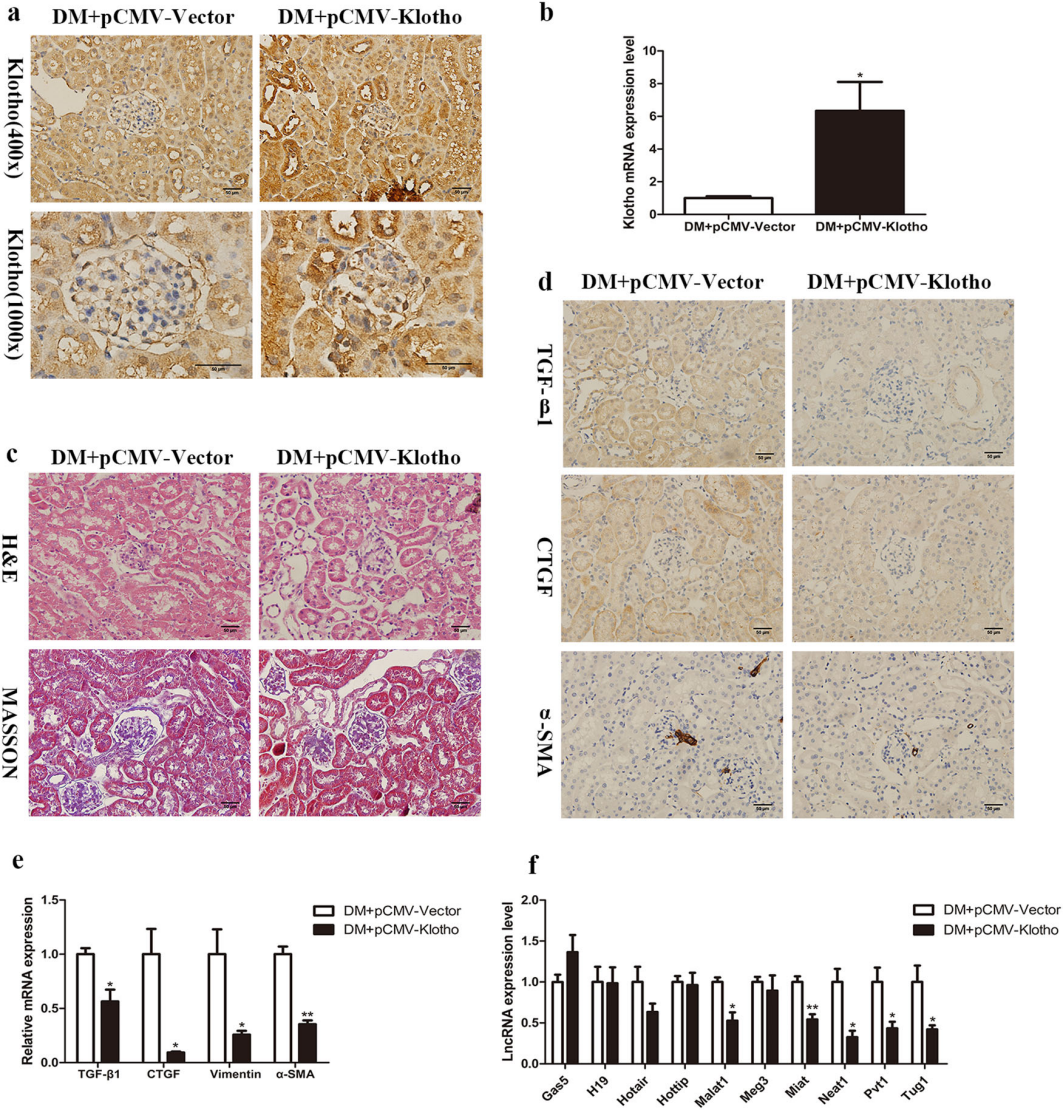
Klotho represses lncRNA Neat1 expression in DM mice. Diabetic model mice established with HFD and STZ induction were transfected with Klotho plasmids by hydrodynamic transfection in vivo. **a**, **b** Immunohistochemistry and qRT-PCR were performed to determine the Klotho expression level in the renal tissues of Klotho-overexpressing DM mice. **c** H&E staining and Masson’s trichrome staining were performed to investigate the protective effect of Klotho on the kidneys of diabetic mice (magnification, ×400). **d**, **e** Immunohistochemistry and qRT-PCR were performed to determine the TGF-β1, CTGF, vimentin and α-SMA expression levels in Klotho-overexpressing DM mice (magnification, ×400). **f** qRT-PCR was performed to determine the levels of long noncoding RNAs (lncRNAs) in Klotho-overexpressing DM mice. Values are the mean ± SEM. *p < 0.05 vs DM + pCMV-vector, **p < 0.01 vs DM + pCMV-vector, ***p < 0.001 vs DM + pCMV-vector.

To explore whether Klotho regulates the expression of lncRNAs during the progression of DKD, a diabetic mouse model established with HFD+STZ induction was transfected with Klotho plasmids by hydrodynamic transfection in vivo. The expression of Klotho mRNA and protein in renal tissue was significantly increased, demonstrating that Klotho overexpression in DM mice was successful (Fig. 1a, b). In addition, we investigated the protective effect of Klotho on the kidneys of diabetic mice. Twenty-four hour urinary microalbumin increased significantly by more than 10 times after diabetes and decreased to some extent after overexpression of Klotho (Table 3). Moreover, the Klotho overexpression group exhibited obvious alleviation of the increase in glomerular volume, thickening of the capillary basement membrane, proliferation of the mesangial matrix and vacuolar degeneration of renal tubular epithelial cells in DM mice (Fig. 1c). Furthermore, the expression of the renal fibrosis markers TGF-β1 and CTGF, as well as the EMT markers vimentin and α-SMA, was detected by immunohistochemistry and qRT-PCR (Fig. 1d, e). We detected EMT-related lncRNAs in renal tissues of Klotho-overexpressing DM mice and found that Neat1 was the most significantly inhibited lncRNA (Fig. 1f). These findings suggested that Neat1 may be regulated by Klotho in the kidneys of DM mice.

**Table 3:** Metabolic profile analysis of mouse parameters.

Parameter	Sham	STZ + HFD	DM + pCMV-Vector	DM + pCMV-Klotho
Glucose (mM)	8.85 ± 0.40	20.95 ± 0.85**	18.65 ± 3.40	20.15 ± 4.02
Urine microalbumin (µg/24 h)	8.17 ± 0.92	88.29 ± 8.67**	80.23 ± 8.46	60.25 ± 6.65^#^
Renal weight index (mg/kg)	6.68 ± 0.62	13.73 ± 2.95*	12.33 ± 2.67	13.18 ± 2.49
Body weight (g)	27.31 ± 1.97	23.30 ± 3.47	24.13 ± 2.13	23.81 ± 3.24
CH (mM)	4.07 ± 1.02	6.84 ± 0.97*	6.46 ± 1.03	6.76 ± 0.98
TG (mM)	5.17 ± 0.55	7.55 ± 0.22**	7.06 ± 0.47	6.97 ± 0.62
LDL-C (mM)	2.39 ± 0.41	4.26 ± 0.35**	4.42 ± 0.38	4.06 ± 0.42
Serum Klotho (pg/ml)	12.91 ± 1.17	6.66 ± 0.301**	6.56 ± 1.01	8.86 ± 2.31^#^

Mean ± SEM; *n* *=* 5; *P *<* 0.05 and **P *<* 0.01 versus Sham; ^#^*P* *<* 0.05 versus DM + pCMV-Vector.

### Neat1 is upregulated in the kidneys of HFD- and STZ-induced diabetic mice

**Fig. 2:**
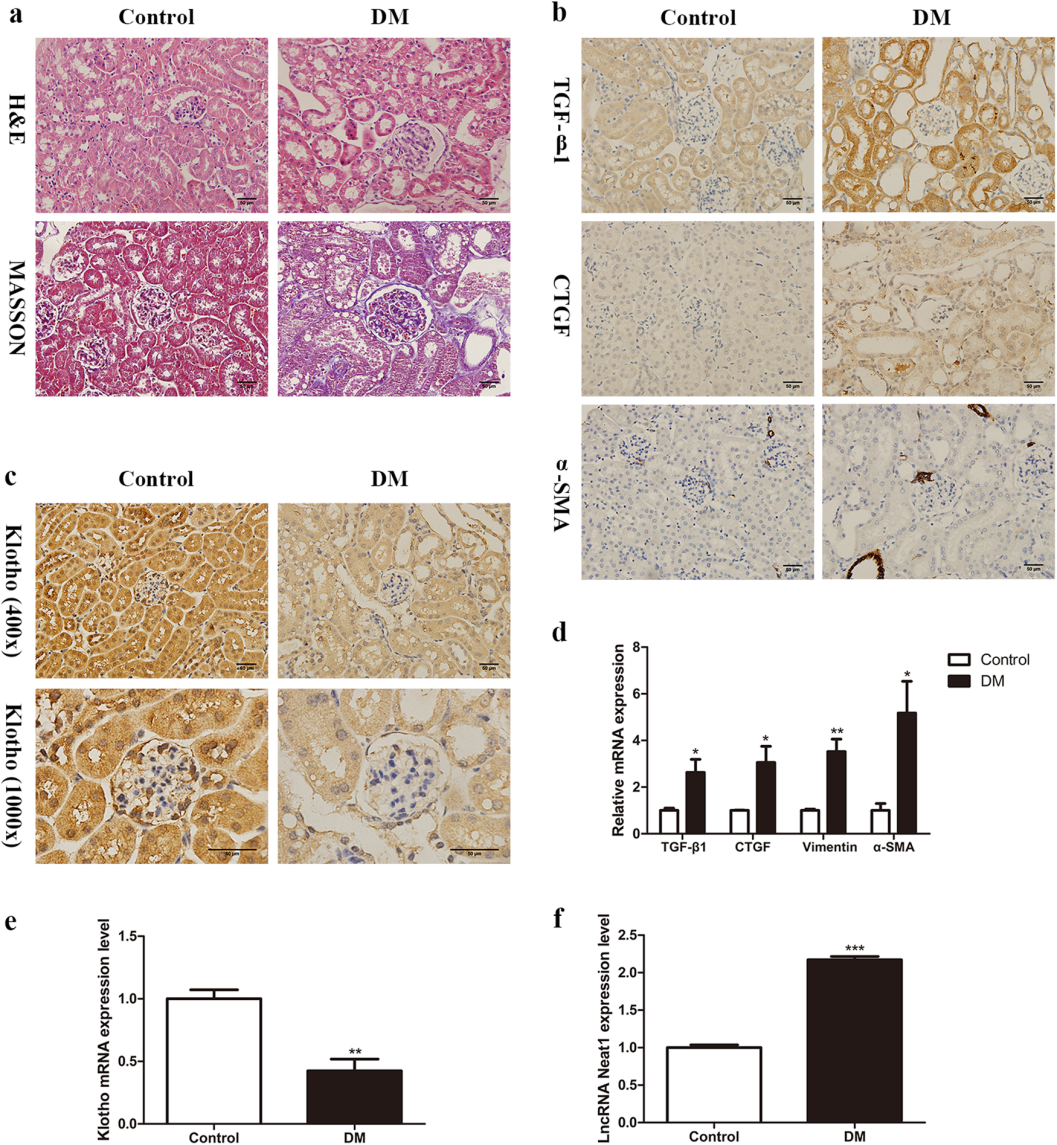
Neat1 is upregulated in HFD- and STZ-induced diabetic mice. **a** H&E staining and Masson’s trichrome staining were performed to investigate whether the DM model group presented typical renal pathological changes of DKD (magnification, ×400). **b**, **d** Immunohistochemistry and qRT-PCR were performed to determine the TGF-β1, CTGF, vimentin and α-SMA expression levels in DM mice (magnification, ×400). **c**, **e** Immunohistochemistry and qRT-PCR were performed to determine the Klotho expression level in DM mice. **f** qRT-PCR was performed to determine the levels of Neat1 in DM mice. Values are the mean ± SEM. *p < 0.05 vs control, **p < 0.01 vs control, ***p < 0.001 vs control.

To determine the correlation between Neat1 and Klotho in DKD, we established a mouse model of HFD combined with STZ-induced DM^17^. Compared to the control group, the DM model group presented typical renal pathological changes of DKD. The glomerular volume of the model group mice increased, and the mesangial matrix proliferated significantly, with obvious vacuole formation in renal tubular epithelial cells (Fig. 2a). The expression of TGF-β1, CTGF, vimentin, and α-SMA was also increased significantly in the kidneys of DM mice, which confirmed that the mice with DM induced by HFD and STZ exhibited tubulointerstitial fibrosis and EMT (Fig. 2b, d). Immunohistochemistry showed that Klotho expression was significantly decreased in renal tubules and interstitial areas (Fig. 2c). We compared the expression of Klotho and Neat1 in the kidneys of the control group and the DM model group. The qRT-PCR results revealed that the level of Klotho was decreased while the level of Neat1 was increased in DM mice (Fig. 2e, f).

### Bovine serum albumin (BSA) increased NEAT1 expression in HK-2 cells

**Fig. 3:**
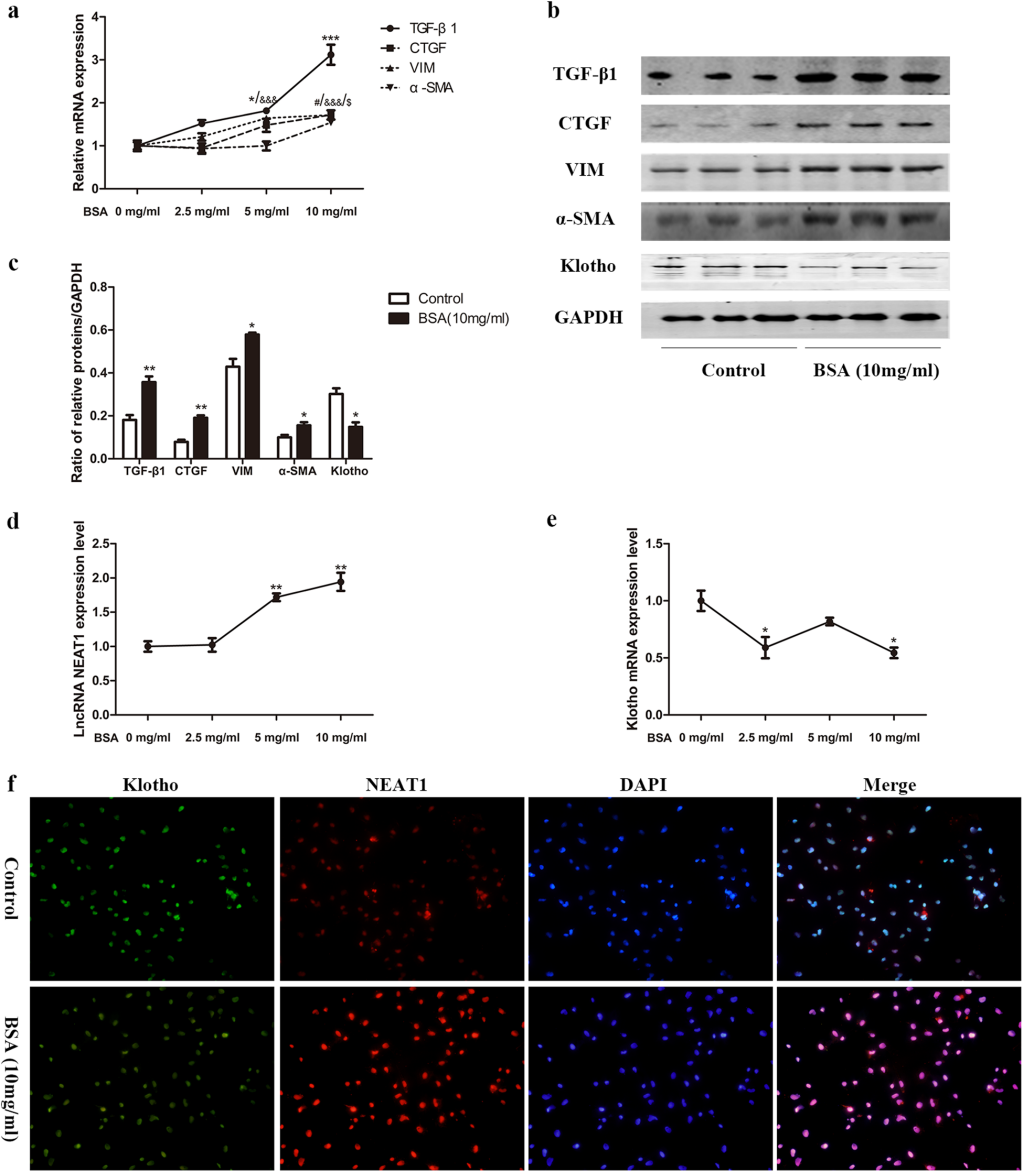
Bovine serum albumin (BSA) upregulated NEAT1 expression in HK-2 cells. HK-2 cells were cultured in different concentrations of BSA. **a** The relative mRNA expression of TGF-β1, CTGF, vimentin and α-SMA in HK-2 cells after incubation with various concentrations of BSA was measured by qRT-PCR. TGF-β1 (*p < 0.05 vs 0 mg/ml, **p < 0.01 vs 0 mg/ml, ***p < 0.001 vs 0 mg/ml); CTGF (^#^*p* < 0.05 vs 0 mg/ml, ^##^*p* < 0.01 vs 0 mg/ml, ^###^*p* < 0.001 vs 0 mg/ml); vimentin (^&^*p* < 0.05 vs 0 mg/ml, ^&&^*p* < 0.01 vs 0 mg/ml, ^&&&^*p* < 0.001 vs 0 mg/ml); α-SMA (^$^*p* < 0.05 vs 0 mg/ml, ^$$^*p* < 0.01 vs 0 mg/ml, ^$$$^*p* < 0.001 vs 0 mg/ml). **b**, **c** The relative protein expression of TGF-β1, CTGF, vimentin, α-SMA, and Klotho in HK-2 cells after incubation with 10 mg/ml BSA was measured by western blots. **d** Relative mRNA expression of Klotho in HK-2 cells after incubation with 10 mg/ml BSA was measured by qRT-PCR. **e** Levels of lncRNA NEAT1 in HK-2 cells after incubation with 10 mg/ml BSA by qRT-PCR. **f** Immunofluorescence and fluorescence in situ hybridization (FISH) were performed to investigate Klotho colocalization with NEAT1 in 10 mg/ml BSA-treated HK-2 cells. *p < 0.05 vs control, **p < 0.01 vs control, ***p < 0.001 vs control.

In our previous work, we found that using human serum albumin culture, HK-2 cells can induce renal tubular cell EMT^6^. Thus, we stimulated HK-2 cells with various concentrations of BSA to mimic DKD in vitro. Treatment with 10 mg/ml BSA significantly increased the levels of TGF-β1, CTGF, vimentin, and α-SMA mRNA (Fig. 3a). Western blot analysis also revealed increased expression of TGF-β1, CTGF, vimentin, and α-SMA and decreased expression of Klotho in the BSA‐treated group (Fig. 3b, c). These data showed that BSA has the capability of inducing fibrosis and EMT in HK-2 cells. Next, we examined the levels of Klotho and NEAT1 using qRT-PCR and found that treating HK‐2 cells with BAS significantly decreased Klotho expression and increased NEAT1 expression (Fig. 3d, e). In addition, Klotho colocalized with NEAT1 in BSA-treated HK-2 cells (Fig. 3f). Taken together, these results demonstrate that the expression of Klotho and NEAT1 showed opposite trends in the in vitro HK-2 cell DKD model.

### NEAT1 promoted BSA-induced HK-2 cell fibrosis and EMT via the ERK1/2 pathway

**Fig. 4:**
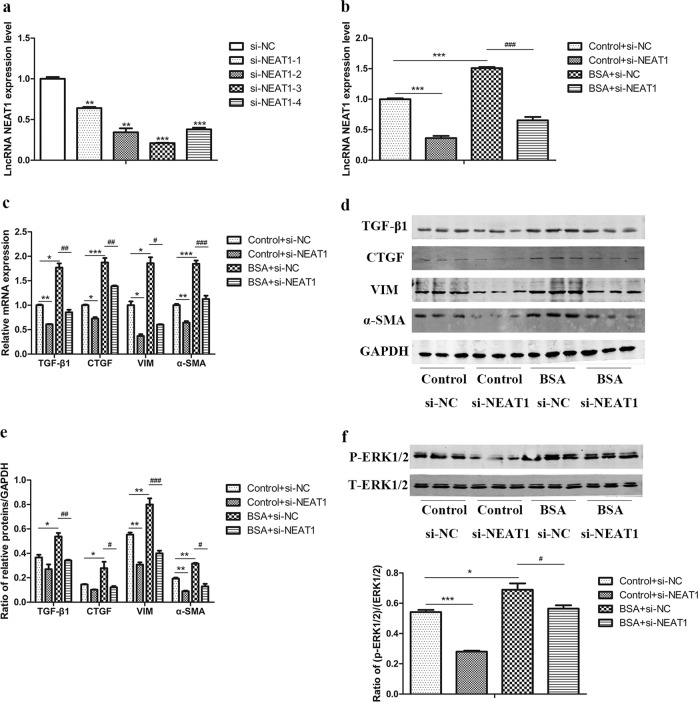
Silencing of NEAT1 inhibited the expression of fibrosis factors and EMT markers in BSA-induced HK-2 cells. **a** qRT-PCR was performed to screen for NEAT1 siRNA in HK-2 cells. *p < 0.05 vs si-NC, **p < 0.01 vs si-NC, ***p < 0.001 vs si-NC. HK-2 cells were transfected with si-NEAT1 and treated with 10 mg/ml BSA for 48 h. **b**, **c** lncRNA NEAT1 expression and the mRNA levels of TGF-β1, CTGF, vimentin, and α-SMA were measured by qRT-PCR. **d**, **e** The protein levels of TGF-β1, CTGF, vimentin, and α-SMA were measured by western blots. **f** Expression of total and phosphorylated ERK1/2 protein was detected by western blots. *p < 0.05 vs control + si-NC, **p < 0.01 vs control + si-NC, ***p < 0.001 vs control + si-NC; ^#^*p* < 0.05 vs BSA + si-NC, ^##^*p* < 0.01 vs BSA + si-NC, ^###^*p* < 0.001 vs BSA + si-NC.

**Fig. 5:**
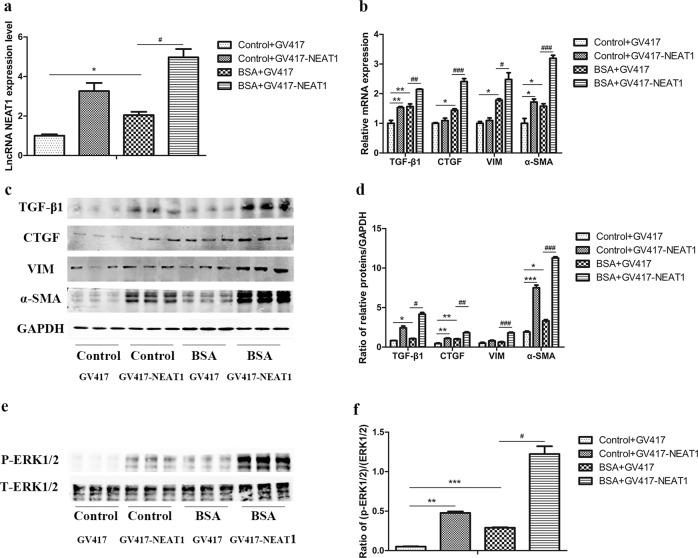
Overexpression of NEAT1 further increased the expression of fibrosis factors and EMT markers in BSA-induced HK-2 cells. **a**, **b** lncRNA NEAT1 expression and the mRNA levels of TGF-β1, CTGF, vimentin, and α-SMA were measured by qRT-PCR. **c**, **d** The protein levels of TGF-β1, CTGF, vimentin, and α-SMA were measured by western blots. **e**, **f** Expression of total and phosphorylated ERK1/2 protein was detected by western blots. *p < 0.05 vs control + GV417, **p < 0.01 vs control + GV417, ***p < 0.001 vs control + GV417; ^#^*p* < 0.05 vs BSA + GV417, ^##^*p* < 0.01 vs BSA + GV417, ^###^*p* < 0.001 vs BSA + GV417.

To investigate whether NEAT1 is involved in fibrosis and EMT in HK-2 cells in vitro, we first screened for the most efficient NEAT1 siRNA (Fig. 4a). Then, we transfected BSA-treated HK-2 cells with si-NEAT1 to observe the changes in fibrosis and EMT in HK-2 cells (Fig. 4b). The results showed that in the absence or presence of BSA, the expression of TGF-β1, CTGF, vimentin, and α-SMA was decreased in cells transfected with si-NEAT1 (Fig. 4c–e). Furthermore, compared to the control cells, BSA-induced HK2 cells transfected with si-NEAT1 exhibited a reversal in cell migration, which is one of the characteristics of EMT in renal tubular cells, as determined through Transwell and wound-healing assays (Supplemental Fig. 1a, b). Therefore, transfection of HK-2 cells with si-NEAT1 can inhibit BSA-induced fibrosis and EMT. In contrast, we transfected BSA-treated HK-2 cells with the GV417-NEAT1 plasmid and found that the overexpression of NEAT1 further increased the expression of TGF-β1, CTGF, vimentin, and α-SMA (Fig. 5a–d). To elucidate the molecular mechanism underlying the effect of NEAT1 on HK-2 cell fibrosis and EMT, we detected the key ERK1/2 signaling pathway components that regulate renal tubular epithelial cell EMT processes^5,6^. We found that silencing or overexpressing NEAT1 expression significantly decreased or increased ERK1/2 phosphorylation (Figs. 4f, 5e, f). In addition, we detected the effects of the ERK inhibitor PD98059 on the expression of these fibrotic factors in BSA-treated HK-2 cells to confirm that the ERK pathway is involved in BSA-induced fibrosis (Supplemental Fig. 3). These data suggested that NEAT1 participates in HK-2 cell fibrosis and EMT partly through the ERK1/2 pathway.

### Overexpression of Klotho suppressed NEAT1 expression in BSA-induced HK-2 cells

**Fig. 6:**
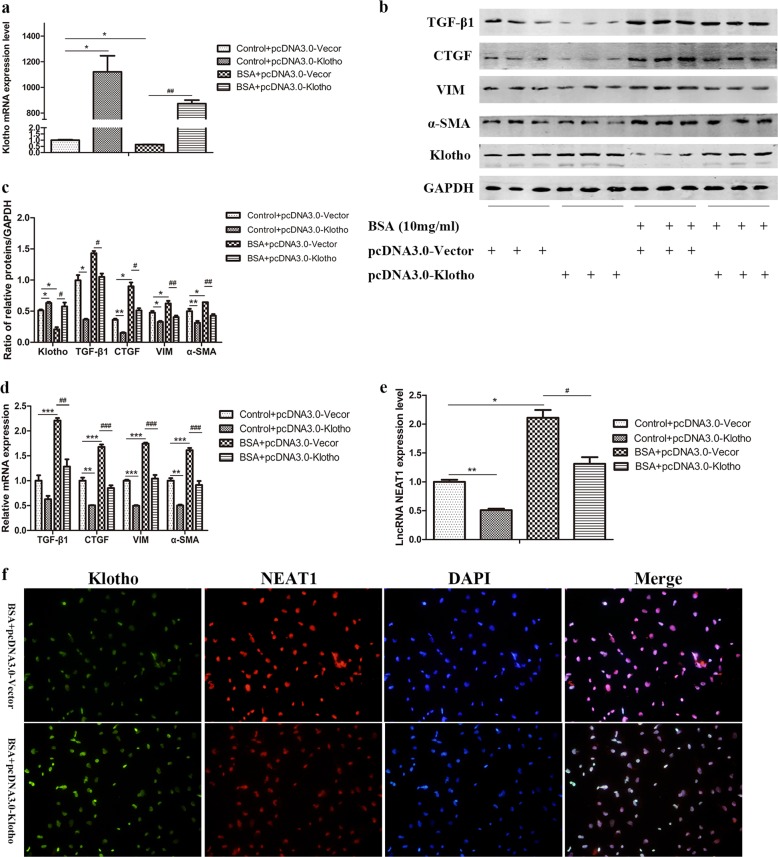
Overexpression of Klotho suppressed NEAT1 expression in BSA-induced HK-2 cells. HK-2 cells were transfected with pcDNA3.0-Klotho and treated with 10 mg/ml BSA for 48 h. **a** Expression of Klotho mRNA was measured by qRT-PCR. **b**, **c** The protein levels of TGF-β1, CTGF, vimentin, α-SMA, and Klotho were measured by western blots. **d**, **e** The mRNA levels of TGF-β1, CTGF, vimentin, α-SMA, and lncRNA NEAT1 were measured by qRT-PCR. **f** Immunofluorescence and FISH were performed to investigate the expression and location of Klotho and NEAT1 after transfection with pcDNA3.0-Klotho in BSA-treated HK-2 cells. *p < 0.05 vs control + pcDNA3.0-vector, **p < 0.01 vs control + pcDNA3.0-vector, ***p < 0.001 vs pcDNA3.0-vector; ^#^*p* < 0.05 vs BSA + pcDNA3.0-vector, ^##^*p* < 0.01 vs BSA + pcDNA3.0-vector, ^###^*p* < 0.001 vs BSA + pcDNA3.0-vector.

To verify the regulatory effect of Klotho on NEAT1 at the cellular level, we transfected BSA-induced HK-2 cells with the pcDNA3.0-Klotho plasmid (Fig. 6a). The protein expression of Klotho was significantly increased, and the expression of TGF-β1, CTGF, vimentin, and α-SMA was decreased after transfection with pcDNA3.0-Klotho in both the absence and presence of BSA (Fig. 6b, c). Klotho overexpression also reduced the increases in fibrosis- and EMT-related mRNA induced by BSA (Fig. 6d). Importantly, Klotho suppressed the increased expression of NEAT1 in BSA-induced HK-2 cells (Fig. 6e). Based on the immunofluorescence and FISH results, NEAT1 expression was also inhibited by Klotho overexpression in BSA-induced HK-2 cells (Fig. 6f). The suppressive effect of Klotho on NEAT1 in BSA-induced HK-2 cells was consistent with that observed in HFD- and STZ-induced DM mice.

### si-Klotho further promoted NEAT1 expression in BSA-induced HK-2 cells

**Fig. 7:**
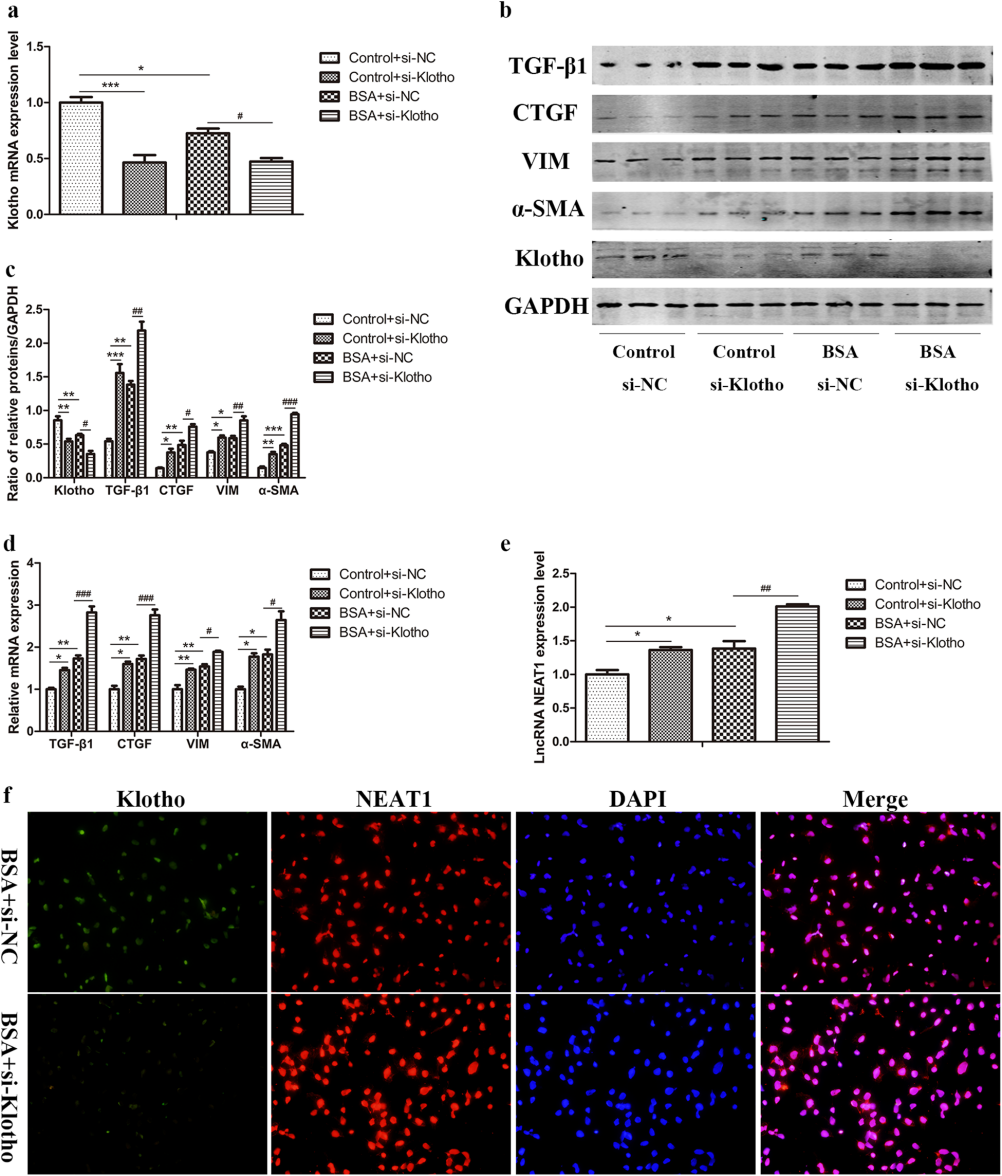
Knockdown of Klotho further promoted NEAT1 expression in BSA-induced HK-2 cells. HK-2 cells were transfected with si-Klotho and treated with 10 mg/ml BSA for 48 h. **a** Expression of Klotho mRNA was measured by qRT-PCR. **b**, **c** The protein levels of TGF-β1, CTGF, vimentin, α-SMA, and Klotho were measured by western blots. **d**, **e** The mRNA levels of TGF-β1, CTGF, vimentin, α-SMA, and lncRNA NEAT1 were measured by qRT-PCR. **f** Immunofluorescence and FISH were performed to investigate the expression and location of Klotho and NEAT1 after transfection with si-Klotho in BSA-treated HK-2 cells. *p < 0.05 vs control + si-NC, **p < 0.01 vs control + si-NC, ***p < 0.001 vs control + si-NC; ^#^*p* < 0.05 vs BSA + si-NC, ^##^*p* < 0.01 vs BSA + si-NC, ^###^*p* < 0.001 vs BSA + si-NC.

In contrast, we transfected BSA-induced HK-2 cells with si-Klotho to verify the regulatory effect of Klotho on the expression of NEAT1 in vitro. The efficiency of si-Klotho was screened in our previous report^13^. The expression of Klotho mRNA was decreased after transfection with si-Klotho in BSA-treated HK-2 cells (Fig. 7a). Western blot analysis showed that si-Klotho significantly upregulated the expression of TGF-β1, CTGF, vimentin, and α-SMA (Fig. 7b, c). Furthermore, the mRNA expression of fibrosis and EMT markers showed the same trends observed for protein expression (Fig. 7d). It was expected that the expression of NEAT1 was further upregulated by silencing Klotho in BSA-induced HK-2 cells (Fig. 7e). Similarly, transfection with si-Klotho in BSA-induced HK-2 cells promoted NEAT1 expression, as observed by immunofluorescence and FISH (Fig. 7f).

### NEAT1 is involved in the protective effect of Klotho

**Fig. 8:**
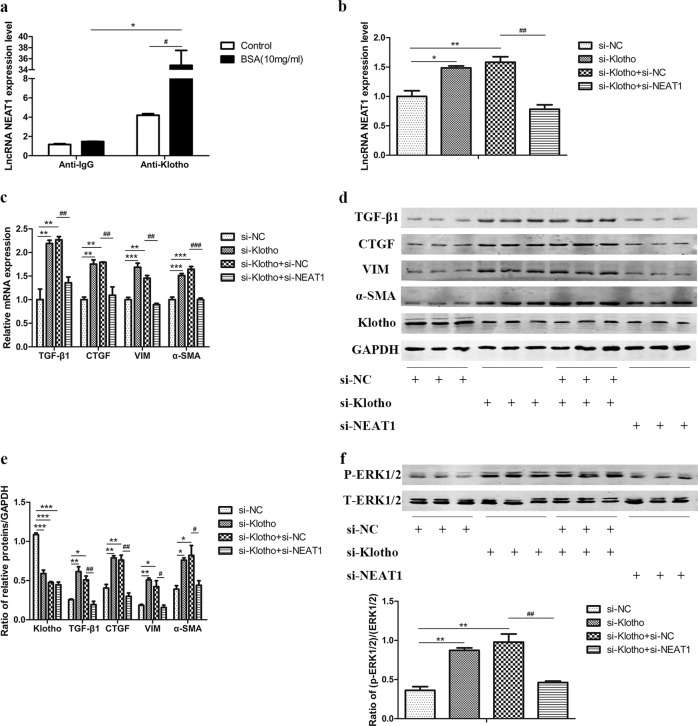
NEAT1 is involved in the protective effect of Klotho. RNA immunoprecipitation was performed using an anti-Klotho antibody or anti-IgG antibody as a control in BSA-treated HK-2 cells for 48 h. **a** lncRNA NEAT1 expression was measured by qRT-PCR. ^*#*^*p* < 0.05 vs control, ^##^*p* < 0.01 vs control, ^###^*p* < 0.001 vs control. HK-2 cells were cotransfected with si-Klotho and si-NEAT1. **b**, **c** lncRNA NEAT1 expression and the mRNA levels of TGF-β1, CTGF, vimentin, and α-SMA were measured by qRT-PCR. **d**, **e** The protein levels of TGF-β1, CTGF, vimentin, α-SMA, and Klotho were measured by western blots. **f** Expression of total and phosphorylated ERK1/2 protein was detected by western blots. *p < 0.05 vs si-NC, **p < 0.01 vs si-NC, ***p < 0.001 vs si-NC; ^#^*p* < 0.05 vs si-Klotho + si-NC, ^##^*p* < 0.01 vs si-Klotho + si-NC, ^###^*p* < 0.001 vs si-Klotho + si-NC.

Since Klotho displays colocalization with NEAT1, we further explored the mechanism by which Klotho regulates NEAT1. We verified that Klotho and NEAT1 interact directly by using the RIP method. The combination of Klotho and NEAT1 was significantly enhanced in BSA-induced HK-2 cells, which may be one of the mechanisms by which Klotho regulates NEAT1 expression (Fig. 8a). Furthermore, we cotransfected si-Klotho and si-NEAT1 into HK-2 cells. We found that silencing NEAT1 eliminated the increases in TGF-β1, CTGF, vimentin, and α-SMA caused by transfection with si-Klotho in HK-2 cells (Fig. 8b–e), as well as the increases in cell migration (Supplemental Fig. 2a, b). In addition, the phosphorylation of ERK1/2 was also inhibited by transfection with si-NEAT1 (Fig. 8f). These results indicate that NEAT1 is involved in the protective effect of Klotho against fibrosis and EMT in renal tubular epithelial cells, partly through the ERK1/2 signaling pathway.

## Discussion

The treatment of DKD remains a challenge for clinicians, despite considerable progress in the development of new antidiabetic and kidney protection drugs^23^. Hence, it is of great significance to understand the potential pathologic mechanism related to DKD for the development of novel therapeutic strategies. Recent studies have reported that the prevention of Klotho decline and direct supplementation of soluble Klotho are both associated with attenuated renal fibrosis and slowed chronic kidney disease (CKD) progression^24^. However, the effect of Klotho in DKD needs further study.

In the current study, we provide evidence that Klotho alleviates renal tubulointerstitial fibrosis and renal tubular EMT in HFD- and STZ-induced DM mice. We used hydrodynamic transfection, which has been effectively used to transfect mice with plasmids in recent decades, to overexpress Klotho in our DM mice^18^. A previous study found that Klotho deficiency exacerbated STZ-induced increases in urine albumin, blood urea nitrogen, expansion of the mesangial matrix in renal glomeruli and kidney hypertrophy^10^. Recently, researchers further demonstrated that hybridization of Klotho transgenic mice significantly attenuated renal hypertrophy, albuminuria, glomerular mesangial expansion, and endothelial glycocalyx loss in spontaneously diabetic Ins2Akita (AKITA) mice^25^. In addition, Klotho has been reported to reduce the expression of fibrotic and inflammatory factors in rat mesangial cells^26,27^. In another study, Klotho also attenuated high-glucose-induced fibronectin and cell hypertrophy in rat renal interstitial fibroblasts^28^. However, the effect of Klotho on renal lncRNA expression has not been reported previously. Therefore, we sought to reveal a new mechanism by which Klotho inhibits the progression of DKD. Specifically, Klotho downregulated the expression of lncRNA NEAT1 to delay the progression of renal tubulointerstitial fibrosis and renal tubular EMT in DKD.

lncRNAs are defined as transcripts longer than 200 nucleotides that are produced by RNA polymerase II and lack protein-coding potential but exert a wide variety of biological functions, and their aberrant expression is associated with diverse pathologies, including cancer and cardiac, neurological, and metabolic diseases^29^. In recent years, some lncRNAs have been found to be related to the development of DKD, but relevant studies are still in the early stages. In this study, we found that the expression of NEAT1 was significantly increased in both HFD- and STZ-induced DM mice and a BSA-induced fibrosis and EMT model in HK-2 cells. It has been reported that proteinuria can promote the progression of renal tubulointerstitial fibrosis and DKD^30,31^. BSA has been reported to induce renal proximal tubular cells to mimic tubulointerstitial fibrosis in vitro^32^. Furthermore, BSA directly decreases Klotho expression in cultured tubular cells, which may explain the decrease in Klotho observed in preclinical and clinical proteinuric kidney disease^33^. Therefore, BSA was used to stimulate HK-2 cells to observe the regulatory effect of Klotho on NEAT1. In the present study, we found that the expression of Klotho showed a pattern opposite to that of NEAT1 and that Klotho colocalized with NEAT1 in BSA-induced HK-2 cells.

NEAT1 has been reported as a long noncoding RNA and functions as one of the main components in the formation and maintenance of paraspeckles^34^. NEAT1 ncRNA is transcribed from a genetic locus called familial tumor syndrome multiple endocrine neoplasia (MEN) type I on human chromosome 11 and comprises two isoforms, 3.7-kb NEAT1_1 and 23-kb NEAT1_2. Both RNAs are produced from the same promoter^35^. Recently, researchers have demonstrated that the FOXN3-NEAT1-SIN3A complex promotes EMT and invasion of breast cancer cells in vitro, as well as dissemination and metastasis of breast cancer in vivo^36^. NEAT1 has also been reported to promote tumor cell EMT, migration, and invasion capacities by stimulating the activation of HIF-2α in hepatocellular carcinoma^37^. In addition, Neat1 aggravated myocardial ischemia-reperfusion injury during diabetic rat cardiomyocyte autophagy by upregulating Foxo1 expression to increase hypoxia-reoxygenation injury^38^. Therefore, to explore the role of NEAT1 in the development of DKD, we transfected si-NEAT1 or the overexpression plasmid GV417-NEAT1 into BSA-induced HK-2 cells and found that the expression of fibrosis and EMT markers was significantly altered. Our results suggested that changing the expression of NEAT1 can regulate renal tubulointerstitial fibrosis and EMT during the development of DKD.

We further explored the mechanism by which NEAT1 exerts its function through the ERK1/2 pathway in BSA-induced HK-2 cells. ERK1/2 is one of the most important components of EGFR activation downstream of the ERK signaling pathway in renal proximal tubules and induces tubulointerstitial fibrosis^39^. Some studies have provided evidence that inhibition of the ERK pathway can ameliorate renal interstitial fibrosis by suppressing tubular EMT^40^. In addition, splice variants of NEAT1 have been reported to be ERK dependent in vivo^41^. To explore the relationship between NEAT1 and the ERK pathway in DKD, we measured the phosphorylation levels of ERK1/2 in transfected HK-2 cells with si-NEAT1 in the absence or presence of BSA. We found that inhibiting the expression of NEAT1 not only directly inhibits the phosphorylation of ERK1/2 but also reverses the increased phosphorylation of ERK1/2 induced by BSA stimulation in HK-2 cells. Furthermore, we detected the effects of the ERK inhibitor PD98059 on the expression of these fibrotic factors in BSA-treated HK-2 cells to confirm that the ERK pathway is involved in BSA-induced fibrosis. Taken together, our results imply that NEAT1 regulates renal tubulointerstitial fibrosis and EMT partly via the ERK1/2 pathway.

To investigate whether NEAT1 is involved in the protective effect of Klotho against renal tubulointerstitial fibrosis and EMT in DKD, we overexpressed and inhibited Klotho and measured the expression of NEAT1 in BSA-induced HK-2 cells. As shown in Figs. 6 and 7, overexpression of Klotho alleviated BSA-induced NEAT1 high expression, while inhibition of Klotho accelerated BSA-induced NEAT1 high expression. Furthermore, we observed that colocalization between Klotho and NEAT1 also existed during BSA-induced Klotho overexpression or inhibition in HK-2 cells. Thus, to verify that Klotho binds to and regulates NEAT1 in DKD, we performed RIP and detected the expression of NEAT1 in BSA-induced HK-2 cells. The combination of Klotho and NEAT1 significantly increased in BSA-induced HK-2 cells to mimic DKD in vitro. Furthermore, cotransfection with si-Klotho and si-NEAT1 significantly inhibited the expression of fibrosis and EMT markers and reduced ERK1/2 phosphorylation, providing further evidence that NEAT1 is involved in the protective effect of Klotho in DKD partly through the ERK1/2 pathway.

In conclusion, this research provides evidence that NEAT1 is involved in the protective effect of Klotho against renal tubulointerstitial fibrosis and EMT in renal tubular epithelial cells through the ERK1/2 signaling pathway in diabetic kidney disease. The correlation between Klotho and NEAT1 provides novel insight into the mechanism by which Klotho may be used to treat diabetic kidney disease.

## Supplementary information

Supplement figure1, 2, 3, figure legends
