# Supplementary material for: Long noncoding RNA NEAT1 is involved in the protective effect of Klotho on renal tubular epithelial cells in diabetic kidney disease through the ERK1/2 signaling pathway
Source: Exp Mol Med. 2020 Feb 14;52(2):266–80. doi: 10.1038/s12276-020-0381-5 (PMC7062691; doi:10.1038/s12276-020-0381-5)
Supplement: Supplementary file 1 — Supplement figure1, 2, 3, figure legends [file 12276_2020_381_MOESM1_ESM.pdf]

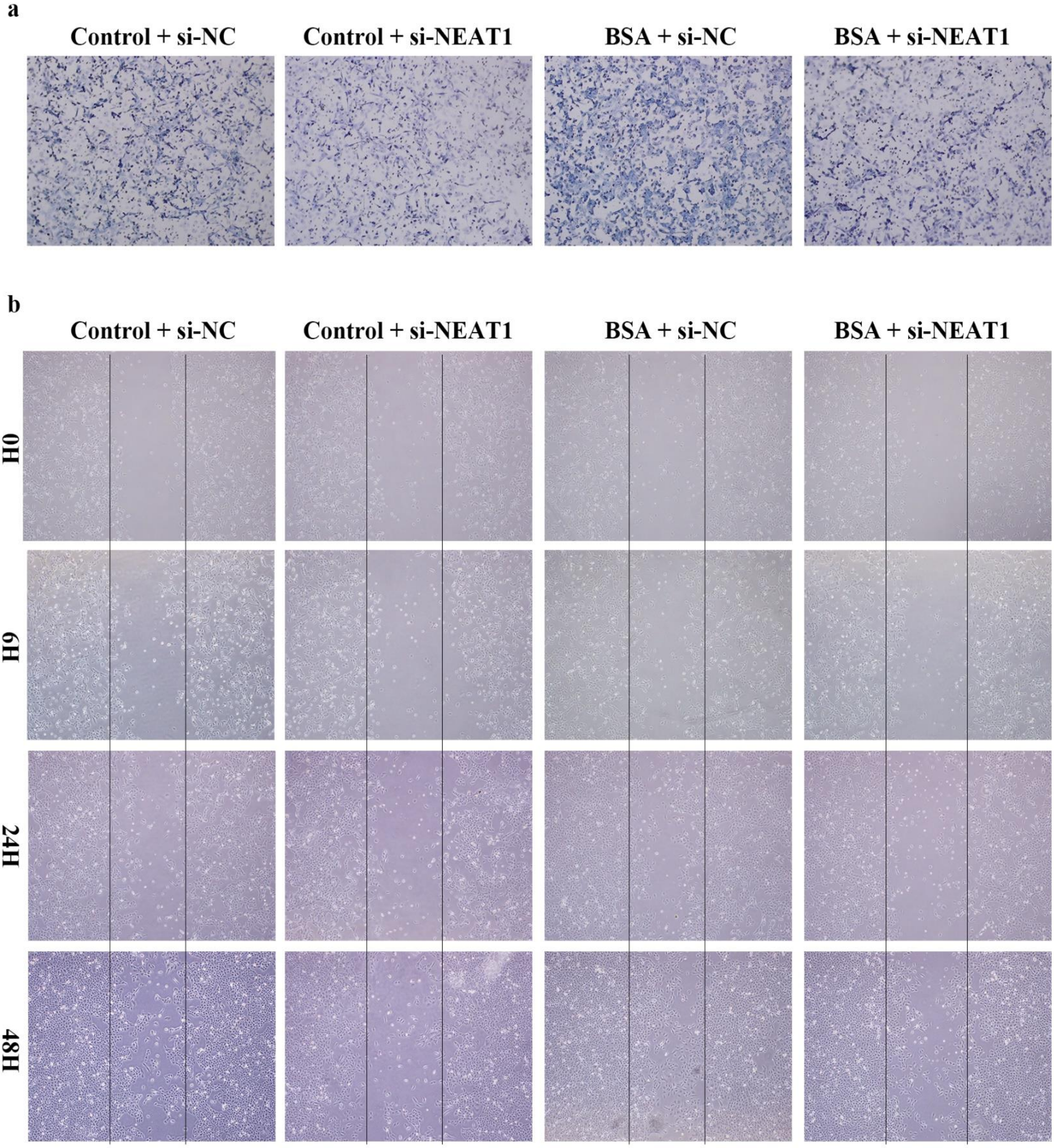
a
Control + si-NC
Control + si-NEAT1
BSA + si-NC
BSA + si-NEAT1
b
Control + si-NC
Control + si-NEAT1
BSA + si-NC
BSA + si-NEAT1
0H
6H
24H
48H

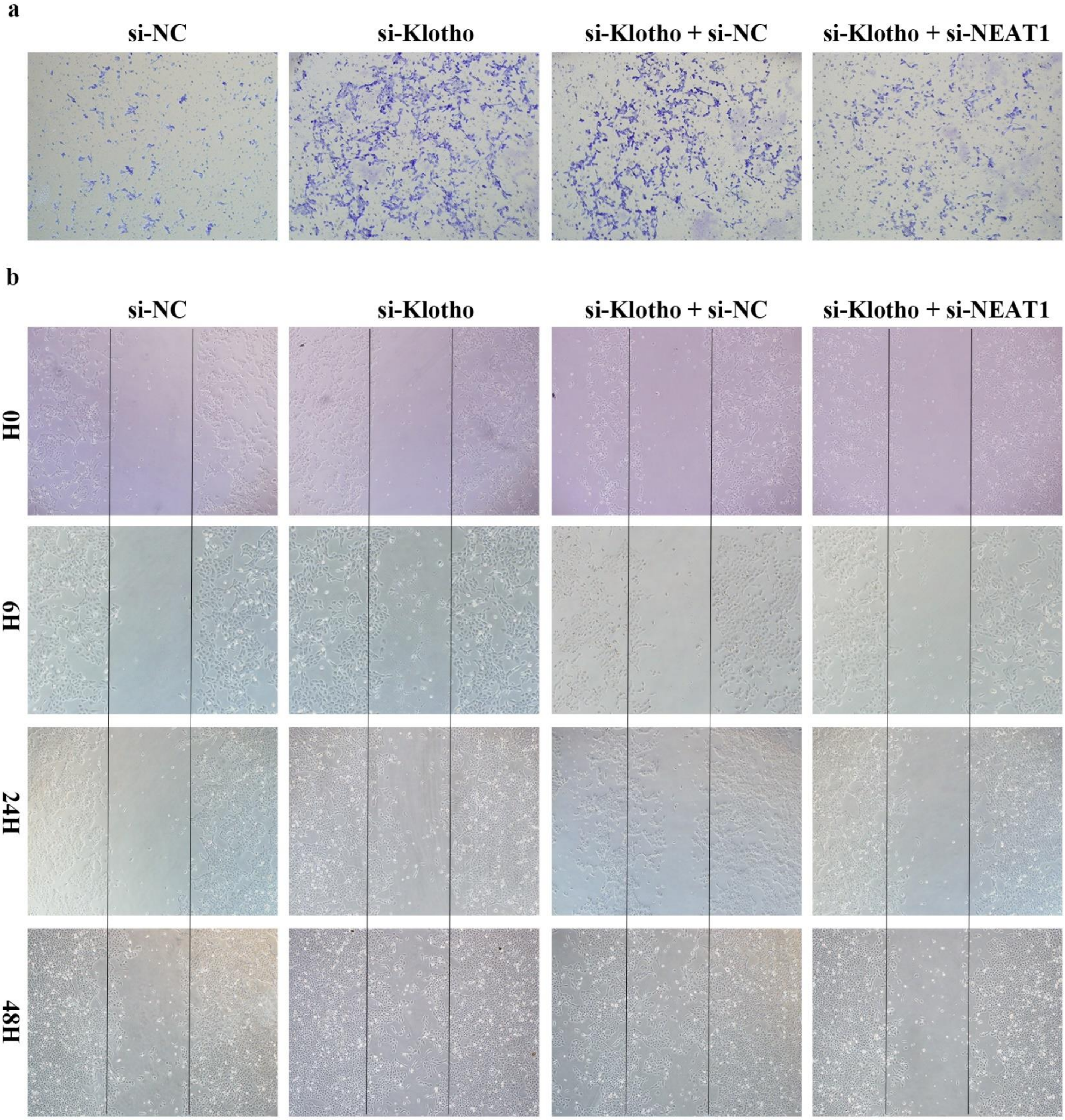

a
si-NC
si-Klotho
si-Klotho + si-NC
si-Klotho + si-NEAT1
b
si-NC
si-Klotho
si-Klotho + si-NC
si-Klotho + si-NEAT1
0H
6H
24H
48H

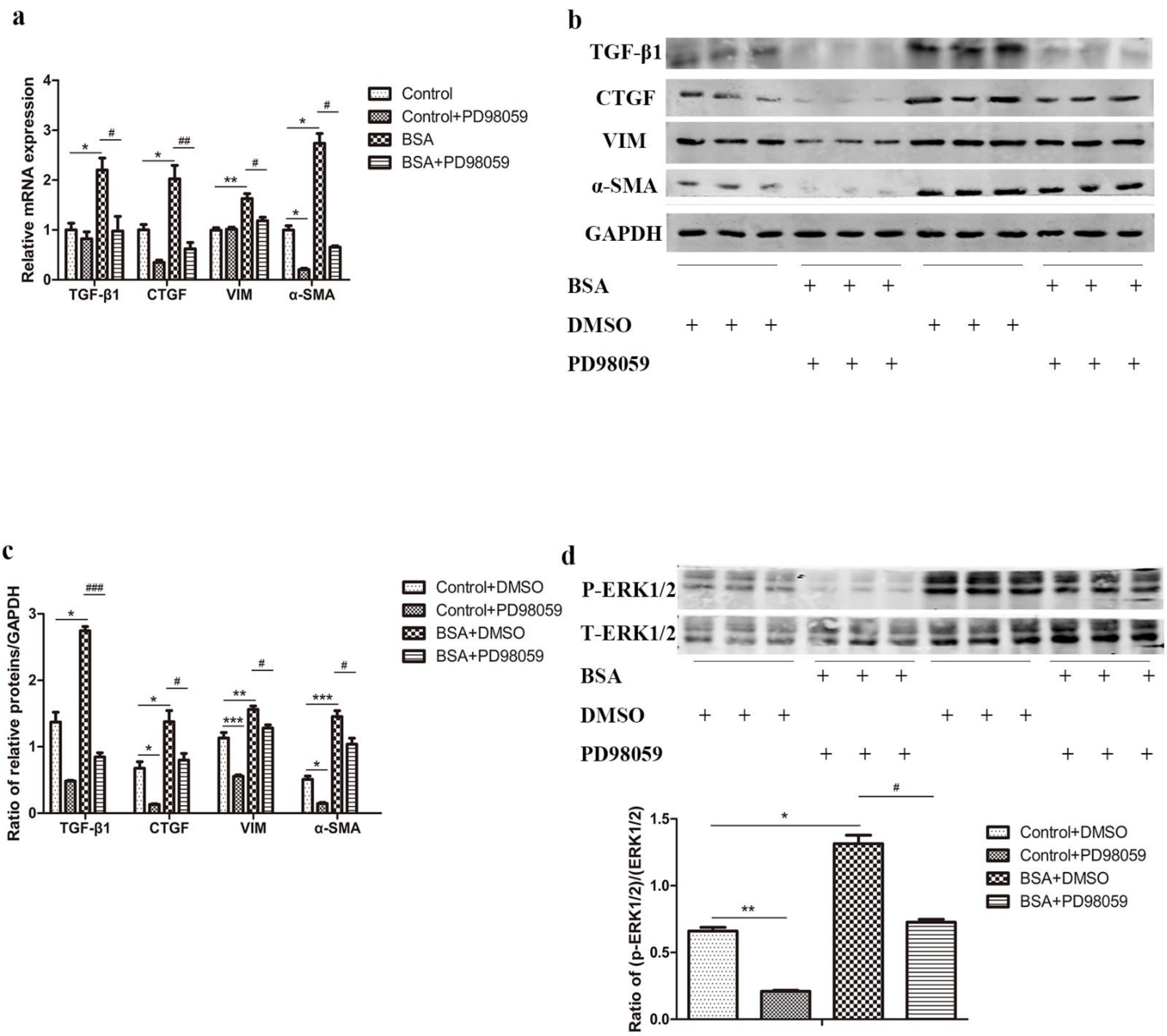
a
Relative mRNA expression
TGF-β1
CTGF
VIM
α-SMA
Control
Control+PD98059
BSA
BSA+PD98059
b
TGF-β1
CTGF
VIM
α-SMA
GAPDH
BSA
DMSO
PD98059
c
Ratio of relative proteins/GAPDH
TGF-β1
CTGF
VIM
α-SMA
Control+DMSO
Control+PD98059
BSA+DMSO
BSA+PD98059
d
P-ERK1/2
T-ERK1/2
BSA
DMSO
PD98059
Ratio of (p-ERK1/2)/(ERK1/2)
Control+DMSO
Control+PD98059
BSA+DMSO
BSA+PD98059

Supplemental figure legends

Supplemental figure 1 NEAT1 promoted BSA-induced EMT in HK-2 cells. The ability of HK-2 cells to migrate was evaluated by Transwell and wound healing assays. (a) HK-2 cells were transfected with si-NEAT1, treated with 10 mg/ml BSA for 24 h and then seeded into the upper chambers in 200 μl of serum-free medium, and 800 μl of 10% fetal calf serum containing medium was used as a chemoattractant in the lower chamber. After incubation for 24 h, the cells that migrated onto the bottom of the filter were fixed with 4% paraformaldehyde and stained with 0.1% crystal violet (Beyotime, Shanghai, China). The cells were then washed and photographed using an inverted microscope (Olympus IX71; Olympus, Tokyo, Japan) at 100x magnification. (b) HK-2 cells were transfected with si-NEAT1 and treated with 10 mg/ml BSA, and scratch wounds were made across the monolayer with the tip of a pipette. Subsequently, the wounded cultures were visualized in serum-free medium at 0, 6, 24 and 48 h, and images (100x) were then captured by inverted microscopy (Olympus IX71; Olympus, Tokyo, Japan) to detect migratory ability.

Supplemental figure 2 NEAT1 is involved in the inhibitory effect of Klotho on EMT in HK-2 cells. The ability of HK-2 cells to migrate was evaluated by Transwell and wound healing assays. (a) HK-2 cells were cotransfected with si-Klotho and si-NEAT1 for 24 h and then incubated in a Transwell chamber for 24 h. The cells were photographed using an inverted microscope (Olympus IX71; Olympus, Tokyo, Japan) at 100x magnification. (b) HK-2 cells were cotransfected with si-Klotho and si-NEAT1, and scratch wounds were made across the monolayer with the tip of a pipette. Subsequently, the wounded cultures were visualized in serum-free medium at 0, 6, 24 and 48 h, and images (100x) were captured by inverted microscopy (Olympus IX71; Olympus, Tokyo, Japan) to detect migratory ability.

Supplemental figure 3 The ERK pathway is involved in BSA-induced fibrosis and EMT in HK-2 cells. (a) The mRNA levels of TGF-β1, CTGF, Vimentin and α-SMA were

measured by qRT-PCR. (b, c) The protein levels of TGF-β1, CTGF, Vimentin and α-SMA were measured by Western blots. (d) Expression of total and phosphorylated ERK1/2 protein was detected by Western blots. *$p < 0.05$ vs Control + DMSO, **$p < 0.01$ vs Control + DMSO, ***$p < 0.001$ vs Control + DMSO; #$p < 0.05$ vs BSA+ DMSO, ##$p < 0.01$ vs BSA + DMSO, ###$p < 0.001$ vs BSA + DMSO.
